# Pretravel consultation on COVID-19 testing before international travel: lessons learnt from the Thai Travel Clinic, Bangkok, Thailand

**DOI:** 10.1186/s40794-021-00139-1

**Published:** 2021-05-29

**Authors:** Wasin Matsee, Phimphan Pisutsan, Watcharapong Piyaphanee

**Affiliations:** 1 Travel Medicine Research Unit, Department of Clinical Tropical Medicine, Faculty of Tropical Medicine, Mahidol University, 420/6 Ratchavithi Road, Bangkok, 10400 Thailand; 2 Thai Travel Clinic, Hospital for Tropical Diseases, Faculty of Tropical Medicine, Mahidol University, Bangkok, Thailand

**Keywords:** COVID-19 test, Certificates, International travel, Pretravel consultation, SARS CoV-2, Air travel

## Abstract

The demand for COVID-19 testing has been on the rise after many countries have eased travel restrictions for essential travel to aid their economy. Thus, we discuss our lessons learnt of the crucial points that need to be considered by the clinicians when dealing with individuals seeking COVID-19 testing before international travel.

## Background

The coronavirus disease 2019 (COVID-19) pandemic has disrupted air travel because of the need for public health measures to contain the spread of the virus. Most countries have sealed their international borders, resulting in a substantial decline in international travel by > 70% in 2020, representing the worst-ever year recorded in the history of tourism [1]. Although several countries have now eased travel restrictions for essential travel to aid their economy [2], some regulations including COVID-19 testing before departure have been implemented to ensure safety during air travel and minimize the risk of COVID-19 transmission. These regulations keep changing, depending on the situation of the country of destination.

## Main text

**Fig. 1:**
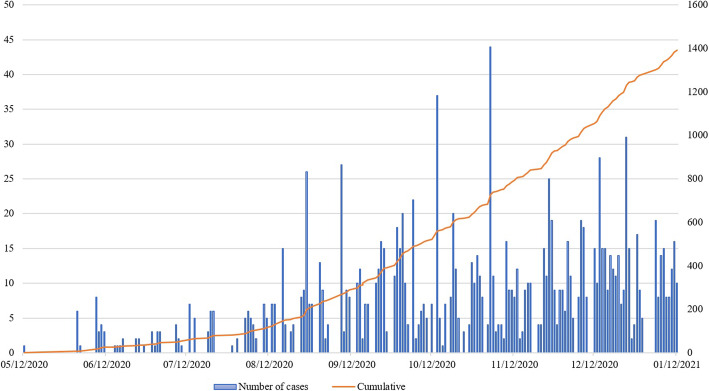
Number of COVID-19 testing before international travel during May 12, 2020 – Jan 12, 2021 at the Thai Travel Clinic, Hospital for Tropical Diseases, Bangkok, Thailand

Our travel clinic is the largest one in Thailand and issues medical certificate for travel requirements or visa application clarifying the COVID-19 risk. We also perform COVID-19 testing since May 2020. People traveling in these circumstances can be provided further counseling in person or via teleconsultation. The testing and medical certificates are issued under medical consideration and as per the airline/country-specific regulations. As of January 12, 2021, 1392 travelers requested COVID-19 testing before international travel. The demand for this test has been increasing continuously from one in May 2020 to > 300 in December 2020 (Fig. 1).

Clinicians who are unfamiliar with these conditions may be unsure of how to approach this group of travelers. Certain important points need to be considered while dealing with these individuals. Here, we discuss a practical approach which we have learnt after providing pretravel consultation on COVID-19 testing service. It can be adopted by clinicians for this purpose.

### Screening point

First, the hospital or clinic should have a screening point to separate individuals who are suspected or at risk of COVID-19. However, asymptomatic individuals may also carry the virus [3]. Thus, health care personnel must ensure proper use of personal protective equipment. Crucial public health and safety measures such as the use of mask, face shield, hand washing and designated area for social distancing for all travelers even they do not have COVID-19 related symptoms.

### Check airline- and country-specific requirements

Clinicians should consciously keep themselves updated with the requirements because the rules keep changing as per the situation. The following issues should be considered:

- **Type of COVID-19 testing**: There are three primary COVID-19 tests—molecular (mostly real-time polymerase chain reaction or RT-PCR), antigen, and antibody tests [4]. Clinicians should ensure that the right test is prescribed with regard to the airline- and country-specific requirements.
- **Timing of the test**: There are specific times for testing before travel. Many airlines or countries require testing and certificate < 48–72 h before the scheduled flight. Thus, clinicians should inquire about the exact date and time of flight departure as well as the name of the airline and lay over time of the flight to evaluate the appropriate date and time of testing. The turnaround time of the test result may also be a key factor.

### Medical assessment before testing

Many airlines and countries require molecular test, and some countries even specify the RT-PCR test. Thus, the nasopharyngeal and throat swab remain the standard procedures for specimen collection. Although, there is no specific contraindication for collecting nasopharyngeal swab specimens, clinicians should be cautious if the individual has had recent nasal trauma or surgery, a markedly deviated nasal septum, or a history of chronically blocked nasal passages or severe coagulopathy [5].

### Risk assessment for other relevant travel-related health risks

Although, many travelers request for COVID-19 testing before travel, clinicians still need to consider other travel-related health measures for specific destinations, such as the administration of yellow fever vaccine and malaria prophylaxis, other preventive measures, and travel health advice. Thus, standard pretravel risk assessments are still needed to ensure traveler safety.

### Issuing of medical certificates

The medical certificate should contain important information including personal information (i.e., name, date of birth, nationality, and passport number), COVID-19 related symptoms on the date and time of examination, type of COVID-19 testing, date and time of specimen collection, date and time of issuing the certificate, and the test result.

Moreover, clinician should advise that a negative testing does not mean individuals will not be exposed to COVID-19 or will not develop it in the future. It is still important to follow social distancing guidelines, practice good hand hygiene, wear a mask, and monitor for signs of illness while traveling and after arrival.

## Conclusion

The demand for pretravel consultation on COVID-19 testing before international travel has increased during the COVID-19 pandemic. Clinicians should understand how to approach the patient and keep themselves updated on the pandemic situation as well as airline- and country-specific requirements.

## Data Availability

The datasets used and/or analyzed during the current study are available from the corresponding author on reasonable request.

## Abbreviations

COVID-19: Corona Virus Disease of 2019
RT-PCR: Real time polymerase chain reaction
SARS-CoV-2: Severe acute respiratory syndrome coronavirus 2
